# Amphiphilic Dendronized Copolymer-Encapsulated Au, Ag and Pd Nanoparticles for Catalysis in the 4-Nitrophenol Reduction and Suzuki–Miyaura Reactions

**DOI:** 10.3390/polym16081080

**Published:** 2024-04-12

**Authors:** Fangfei Liu, Xiong Liu

**Affiliations:** State Key Laboratory of Chemistry and Utilization of Carbon Based Energy Resources, College of Chemistry, Xinjiang University, Urumqi 830017, China; liufangfei1214@163.com

**Keywords:** amphiphilic dendronized copolymer, 4-nitrophenol reduction, Suzuki–Miyaura reactions

## Abstract

The branched structures of dendronized polymers can provide good steric stabilization for metal nanoparticle catalysts. In this work, an amphiphilic dendronized copolymer containing hydrophilic branched triethylene glycol moieties and hydrophobic branched ferrocenyl moieties is designed and prepared by one-pot ring-opening metathesis polymerization, and is used as the stabilizer for metal (Au, Ag and Pd) nanoparticles. These metal nanoparticles (Au nanoparticles: 3.5 ± 3.0 nm; Ag nanoparticles: 7.2 ± 4.0 nm; Pd nanoparticles: 2.5 ± 1.0 nm) are found to be highly active in both the 4-nitrophenol reduction and Suzuki–Miyaura reactions. In the 4-nitrophenol reduction, Pd nanoparticles have the highest catalytic ability (TOF: 2060 h^−1^). In addition, Pd nanoparticles are also an efficient catalyst for Suzuki–Miyaura reactions (TOF: 1980 h^−1^) and possess good applicability for diverse substrates. The amphiphilic dendronized copolymer will open a new door for the development of efficient metal nanoparticle catalysts.

## 1. Introduction

Metal nanoparticle catalysts have been widely investigated in homogeneous and heterogeneous catalysis over the past few decades because of their superior catalytic activity derived from their high specific surface area and quantum size effect [1,2]. Along with decreasing the particle size of metal nanoparticles, more active metal atoms are exposed on their surface and can serve as active sites, which will greatly enhance atomic utilization efficiency and improve catalytic performance [3,4]. Consequently, metal nanoparticles with relatively small sizes (<10 nm) often have better catalytic ability compared to large metal nanoparticle catalysts. Nevertheless, metal nanoparticles with small sizes might result in serious aggregation owing to the high surface energy, which would cause a loss or reduction in the catalytic performance [5,6,7,8,9,10]. To solve this problem, great efforts have been made in the past decade. At present, various supporting materials including zeolites, metal–organic frameworks, carbon-based materials, inorganic oxides, covalent organic frameworks and polymers have been reported to immobilize small-sized metal nanoparticles for catalysis [1,2,3,4]. Metal nanoparticles are able to be stabilized via electron interactions or spatial limitation with supporting materials; thus, the synergistic effects between metal nanoparticles and supporting materials can enhance the catalytic ability.

Polymers have been considered as a kind of ideal supporting materials for metal nanoparticle catalysts, because their structures and functionalities are easy to design and all kinds of functional atoms including oxygen, phosphorus, sulfur and nitrogen can be introduced into polymers to interact with metal nanoparticles to control their growth [5]. So far, a variety of polymers such as homopolymers, block copolymers, dendrimers, dendronized polymers, hyperbranched polymers, star polymers, and polymer networks have been developed as supporting materials for metal nanoparticle catalysts. For example, Gao et al. designed hyperbranched polytriazoles to support Pd nanoparticles (ca. 10 nm) for catalyzing the Heck reaction [11]. Xie’s group reported that an amphiphilic star-like β-cyclodextrin-*g*-poly(4-vinyl pyridine)-*b*-polystyrene diblock copolymer was used for controllable synthesis of stable Au nanoclusters (<2 nm) whose stability was proved in the 4-nitrophenol reduction via NaBH_4_ [12]. Peinemann and colleagues found that the cross-linked β-cyclodextrin polymer network could be applied to prepare various metal (Pd, Ag, Au, Pt and Rh) nanoparticles with narrow size distribution, and the obtained Pd nanoparticles might serve as a heterogeneous catalyst for the low-temperature hydrogenation of various nitroarenes and the Suzuki–Miyaura reaction [13]. Indeed, polymers have shown a promising prospect for supporting metal nanoparticle catalysts based on steric stabilization.

Dendronized polymers are a type of typical branched polymer with well-defined dendritic wedges [14,15,16,17,18]. Compared to traditional dendrimers, a significant advantage is that dendronized polymers with a low generation level even possess more branched structures than higher-generation dendrimers [14,15,16,17,18]. The branched structures of dendronized polymers can provide good steric stabilization for metal nanoparticle catalysts [19]. Frechet and coworkers developed the first dendronized polymer with polyester dendrons to create a microenvironment for catalyzing a difficult esterification [17]. In addition, amphiphilic copolymers have the ability to self-assemble into micelles in solution, offering protection and solubilization for metal nanoparticle catalysts and creating a unique molecular microenvironment for various chemical reactions [20,21]. Gu et al. found that a second-generation highly branched amphiphilic dendronized copolymer (ADC) can be used as a stabilizer for Au and Ag nanoparticles, and the resulting metal nanoparticles were efficient catalysts for the 4-nitrophenol reduction [21].

**Figure 1:**
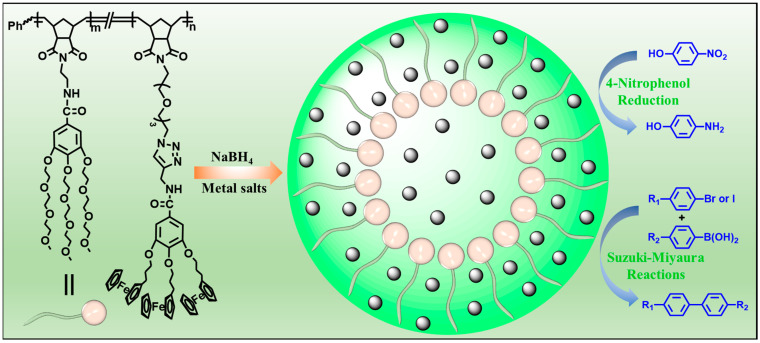
Synthesis of metal (Au, Ag and Pd) nanoparticles for catalysis.

With continuous efforts to develop functional polymers for supporting metal nanoparticle catalysts, an amphiphilic dendronized copolymer (ADC) containing hydrophilic branched triethylene glycol moieties and hydrophobic branched ferrocenyl moieties was readily prepared by one-pot ring-opening metathesis polymerization (ROMP). The ADC polymer was used to support metal (Au, Ag and Pd) nanoparticles (ADC-AuNPs, ADC-AgNPs and ADC-PdNPs) for catalysis (Figure 1). The catalytic properties of these metal nanoparticles were first investigated and compared in the 4-nitrophenol reduction. Furthermore, Suzuki–Miyaura cross-coupling reactions were adopted to further detect the catalytic ability and applicability of ADC-PdNPs.

## 2. Experimental Procedure

### 2.1. Materials

Silver nitrate (AgNO_3_), chloroauric acid (HAuCl_4_), potassium chloride (KCl), potassium carbonate (K_2_CO_3_), Grubbs’ second-generation metathesis catalyst, 4-nitrophenol, dichloromethane (CH_2_Cl_2_), ethyl vinyl ether, sodium borohydride (NaBH_4_), palladium chloride (PdCl_2_) and other chemicals were provided by Energy Chemical (The Woodlands, TX, USA). Grubbs’ 3rd generation catalyst, hydrophilic monomer and ferrocene-containing hydrophobic monomer were synthesized according to previous report [22,23]. All chemicals are of analytical grade and were used directly.

### 2.2. Instruments

^1^H NMR (Bruker AV Ⅱ-400MHz, Munich, Germany) was adopted to characterize the chemical structures of compounds. UV2550 spectrophotometer was used for UV–vis spectra analysis. Transmission electron microscopy (TEM, JEOL JEM-2100 F, Tokyo, Japan) was applied to analyze the microstructures of products. Gel permeation chromatography (GPC, Agilent waters 1515, Santa Clara, CA, USA) analysis was carried out in *N, N*-dimethylformamide, and poly(methyl methacrylate) standards were used.

### 2.3. Synthesis of Amphiphilic Dendronized Copolymer

The ROMP synthesis for amphiphilic dendronized copolymer was from the previous literature [21]. Hydrophilic monomer (500 mg, 0.63 mmol, 60 equiv) and ferrocene-containing hydrophobic monomer (263 mg, 0.21 mmol, 20 equiv) were dissolved in CH_2_Cl_2_ (3 mL). Then, Grubbs’ 3rd generation catalyst (9.3 mg, 0.0105 mmol, 1 equiv) was added into the above solution and the obtained solution was vigorously stirred at room temperature under N_2_ for 8 h. The reaction was quenched with ethyl vinyl ether (0.4 mL). The reaction mixture was concentrated and precipitated with methanol, followed by washing with petroleum ether. An amphiphilic dendronized copolymer with a yellow-brown color was obtained and was dried in vacuum overnight (yield: 95%). ^1^H NMR (400 MHz, CDCl_3_, 25 °C, TMS), δ ppm: 7.78 ppm (triazolyl), 7.07 ppm (phenyl), 5.60 ppm (olefinic), 5.49 ppm (olefinic), 4.65 ppm (CH_2_-triazolyl), 4.50 ppm (CH_2_-triazolyl), 4.16–4.04 ppm (ferrocenyl), 3.81–3.52 ppm (OCH_2_ and NCH_2_CH_2_N), 3.36 ppm (CH_3_), 3.34 ppm (CH_3_), 2.98 ppm (=CH-CH and CH-CO), 2.62–2.52 ppm (OCH_2_CH_2_CH_2_), 1.52 ppm (CH=CHCHCH_2_). GPC data: Mn = 85,834 Da, Mw = 96,298 Da, PDI = 1.12.

### 2.4. Preparation of Amphiphilic Dendronized Copolymer-Encapsulated Au, Ag and Pd Nanoparticles

Metal salt precursors (AgNO_3_, HAuCl_4_ and PdCl_2_) with a concentration of 1 × 10^−3^ mol/L were first prepared in deionized water. Then, the metal salt solution (2 mL) was added to a mixed solution containing deionized water (18 mL), amphiphilic dendronized copolymer (20 mg) and excess NaBH_4_ (2 × 10^−2^ mmol, 10 eq of metal salt precursors), and then the obtained solution was stirred at 30 °C for 2 h. In the experiments, metal salts were reduced by excess NaBH_4_ (a strong reducing agent) to ensure the full reduction process, which was consistent with the previous literature [21]. Meanwhile, no precipitation was generated. A uniform homogeneous solution was obtained. So, the concentration of amphiphilic dendronized copolymer-encapsulated metal nanoparticles (ADC-AuNPs, ADC-AgNPs and ADC-PdNPs) was determined to be 1.0 × 10^−4^ mol/L. For the preparation of the amphiphilic dendronized copolymer-encapsulated Pd nanoparticles, equimolar KCl with respect to PdCl_2_ was supplemented to improve the solubility in water.

### 2.5. General Process for the 4-Nitrophenol Reduction Using Amphiphilic Dendronized Copolymer-Encapsulated Au, Ag and Pd Nanoparticles as Catalysts

Amphiphilic dendronized copolymer-encapsulated Au, Ag and Pd nanoparticles were used to catalyze the 4-nitrophenol reduction with NaBH_4_ as the reductive agent. Concretely, 2.5 mL of deionized water was first used to dissolve 0.06 mg of 4-nitrophenol in a standard quartz cell. Whereafter, 1.6 mg of NaBH_4_ was added into the 4-nitrophenol solution. Then, 2.0 mol% or 0.5 mol% of amphiphilic dendronized copolymer-encapsulated Au, Ag and Pd nanoparticles were quickly placed into the standard quartz cell to catalyze the 4-nitrophenol reduction. The reduction reaction was detected through UV–vis spectra. All the catalytic experiments were performed three times.

### 2.6. General Process for Suzuki–Miyaura Reactions Using Amphiphilic Dendronized Copolymer-Encapsulated Pd Nanoparticles as Catalyst

Aryl halides (1.0 mmol), arylboronic acids (1.1 mmol), amphiphilic dendronized copolymer-encapsulated Pd nanoparticles (10 ppm, 50 ppm, 100 ppm, 200 ppm, 300 ppm, 400 ppm, 500 ppm, respectively) and K_2_CO_3_ (2.0 mmol) were placed into a small tube. Then, EtOH (2.5 mL)/H_2_O (2.5 mL) system was used as the solvent. The obtained mixture was stirred at 85 °C for 10 h. Then, CH_2_Cl_2_ (3 mL) was used to extract the reaction mixture three times. Crude product was obtained by concentrating the collected organic phase and was purified through column chromatography. ^1^H NMR spectrum was adopted to characterize the desired products. All the catalytic experiments were performed three times.

## 3. Results and Discussion

**Figure 2:**
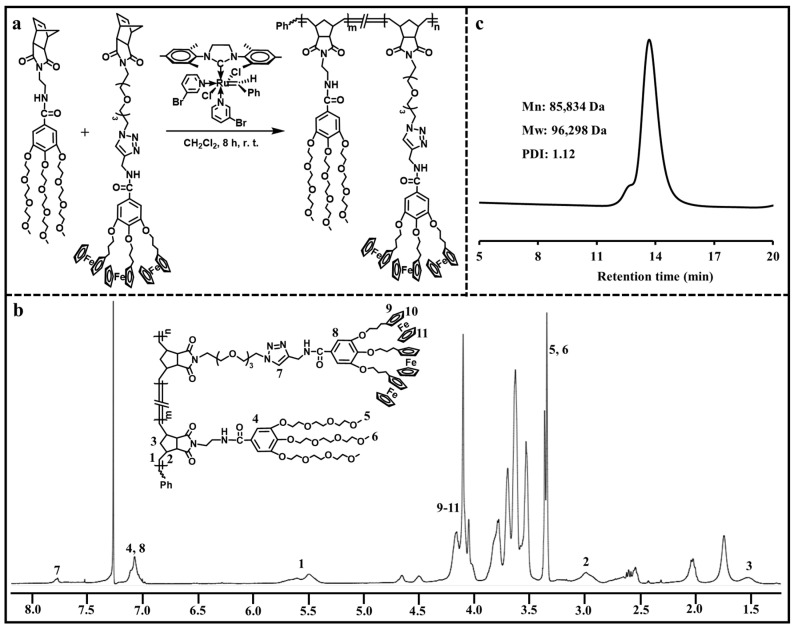
(**a**) Synthesis of the ADC polymer. (**b**) ^1^H NMR spectrum of the ADC polymer. (**c**) GPC curve of the ADC polymer.

ROMP has been considered as a kind of controlled polymerization technique for synthesizing a variety of functional polymers with well-defined structures [24,25,26,27]. Here, ROMP was applied to prepare the ADC polymer containing hydrophilic branched triethylene glycol moieties and hydrophobic branched ferrocenyl moieties. As shown in Figure 2a, ROMP of hydrophilic monomer and ferrocene-containing hydrophobic monomer with the help of Grubbs’ 3rd generation catalyst resulted in the amphiphilic ADC polymer. The chemical structure of the ADC polymer was analyzed via ^1^H NMR (Figure 2b). The typical triazolyl proton peak was found at 7.78 ppm, and the ferrocenyl proton peaks were located at 4.16–4.04 ppm. The peak at 7.07 ppm corresponded to the phenyl protons. The representative double peaks of olefinic protons in poly(norbornene) were observed at 5.60 ppm and 5.49 ppm. The peaks at 3.36 ppm and 3.34 ppm were assigned to the methyl proton peaks in the hydrophilic moieties. These results confirmed that the ADC polymer was synthesized successfully. The molecular weight of the ADC polymer was determined via GPC (Figure 2c). The ADC polymer had an Mn value of 85,834 Da, an Mw value of 96,298 Da and a PDI value of 1.12.

**Figure 3:**
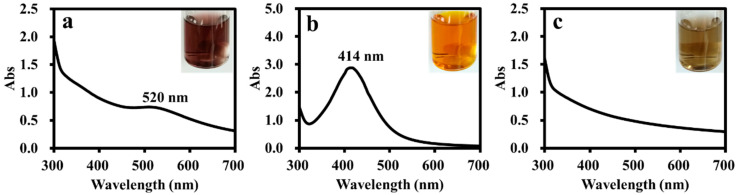
UV–vis. spectra for ADC-AuNPs (**a**), ADC-AgNPs (**b**) and ADC-PdNPs (**c**).

The ADC polymer with branched structures and amphiphilic feature should be a suitable supporting material for metal nanoparticle catalysts. Therefore, the ADC polymer was used as a stabilizer for the preparation of Au, Ag and Pd nanoparticles. Using the metal salt (AgNO_3_, HAuCl_4_ and PdCl_2_) reduction strategy, ADC-AuNPs, ADC-AgNPs and ADC-PdNPs could be synthesized, using the ADC polymer as the stabilizer and NaBH_4_ as the reductive agent. ADC-AuNPs showed a wine-red color, ADC-AgNPs exhibited a yellowish-brown color, and ADC-PdNPs presented a light-brown color (Figure 3). UV–vis spectra analysis manifested that ADC-AuNPs and ADC-AgNPs had a typical surface plasmon band at 520 nm (corresponded to Au^0^, Figure 3a) and 414 nm (corresponded to Ag^0^, Figure 3b), respectively. It is worth noting that ADC-PdNPs had no obvious surface plasmon band (Figure 3c), which was also observed in the previous literature [28,29].

**Figure 4:**
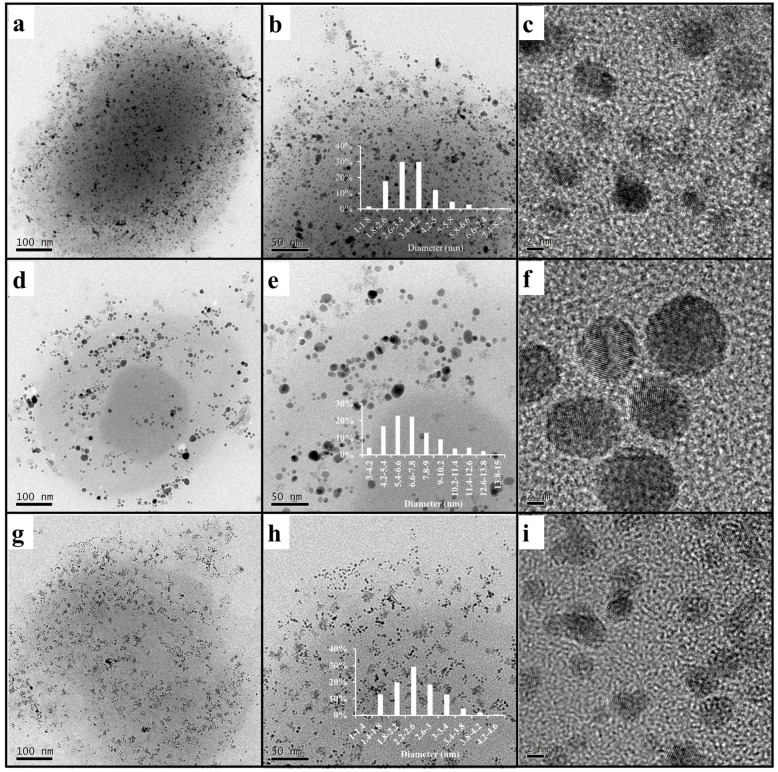
TEM images of ADC-AuNPs ((**a**,**b**), 3.5 ± 3.0 nm), ADC-AgNPs ((**d**,**e**), 7.2 ± 4.0 nm) and ADC-PdNPs ((**g**,**h**), 2.5 ± 1.0 nm), and HRTEM images of ADC-AuNPs (**c**), ADC-AgNPs (**f**) and ADC-PdNPs (**i**). The inserted pictures represent the size distribution plot of the metal NPs.

TEM and HRTEM analyses were adopted to characterize the morphologies and sizes of ADC-AuNPs, ADC-AgNPs and ADC-PdNPs. As can be seen from Figure 4, these metal nanoparticles were quasi-spherical. The average sizes of ADC-AuNPs, ADC-AgNPs and ADC-PdNPs were 3.5 ± 3.0 nm, 7.2 ± 4.0 nm and 2.5 ± 1.0 nm, respectively. In HRTEM images, ADC-AuNPs, ADC-AgNPs and ADC-PdNPs had the clear lattice fringe with interplanar spacing of 0.23 nm, 0.20 nm and 0.23 nm, respectively. As a result, ADC-AuNPs were consistent with the Au (111) plane [30,31,32], ADC-AgNPs were in accordance with the Ag (200) plane [33], and ADC-PdNPs were derived from the Pd (111) plane [34,35]. TEM and HRTEM analyses demonstrated that these metal nanoparticles possessed small sizes, which should be beneficial to catalysis.

**Figure 5:**
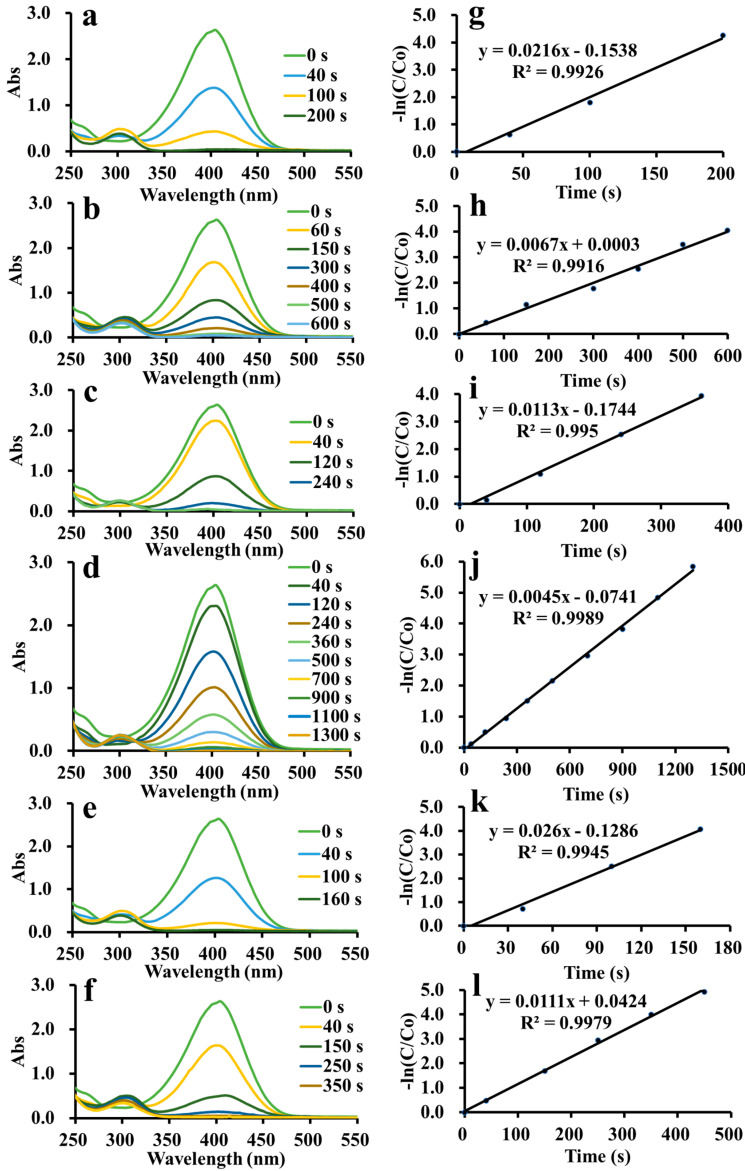
Real-time UV–vis spectra in the reduction of 4-nitrophenol and plots of [−ln(C/C0)] vs. time [ADC-AuNPs: 2.0 mol% (**a**,**g**) and 0.5 mol% (**b**,**h**); ADC-AgNPs: 2.0 mol% (**c**,**i**) and 0.5 mol% (**d**,**j**); ADC-PdNPs: 2.0 mol% (**e**,**k**) and 0.5 mol% (**f**,**l**)].

The catalytic activities of ADC-AuNPs, ADC-AgNPs and ADC-PdNPs were studied in the reduction of 4-nitrophenol. As is well known, the 4-nitrophenol reduction is a representative model reaction used to estimate the catalytic performance of metal nanoparticles [36,37]. Although the 4-nitrophenol reduction is thermodynamically possible, it is kinetically limited without catalysts [38]. A high potential difference between electron donors and electron acceptors would cause a kinetic barrier. Nevertheless, catalysts may serve as the electronic relay to induce the reduction reaction by overcoming the kinetic barrier. In this way, electron transfer would occur from BH_4_^−^ to 4-nitrophenol [39]. The catalytic ability of metal nanoparticles is highly relevant to its high specific surface area. Therefore, electron transfer could induce the reduction reaction. The reduction reaction was easily detected through UV–vis spectra, as shown in Figure 5a–f. 4-Nitrophenol had a characteristic surface plasmon band at 317 nm, which rapidly red-shifted to 400 nm when NaBH_4_ was added to the solution because of the generation of a 4-nitrophenolate ion [40]. The previous literature has demonstrated that the typical surface plasmon band at 400 nm would weaken gradually after adding catalysts due to the consumption of 4-nitrophenol [41,42,43,44]. Meanwhile, a new surface plasmon band of 4-aminophenol would appear in the UV–vis spectra. ADC-AuNPs, ADC-AgNPs and ADC-PdNPs were found to be able to smoothly catalyze the 4-nitrophenol reduction (Figure 5a–f). In the 4-nitrophenol reduction, NaBH_4_ (100 eq) was in high excess relative to 4-nitrophenol; thus, the reaction rate did not depend on NaBH_4_. Therefore, the 4-nitrophenol reduction was regarded as the pseudo-first-order kinetics. The concentration of the 4-nitrophenol was proportional to its absorbance. The concentration at starting (C0) and at time t (C) was considered to be equivalent to the absorbance at starting (A0) and at time t (A). The rate constant (K) could be confirmed by plots of [−ln(C/C0)] vs. reduction time. So, the corresponding K values may be described as follows:

$$-\ln\left(\frac{A}{A0}\right)=-\ln\left(\frac{C}{C0}\right)=Kt$$

As shown in Figure 5g–i, the plots of [−ln(C/C0)] vs. reduction time exhibited a linear dependence, manifesting good pseudo-first-order kinetics. Turnover frequency (TOF) was used to compare the catalytic effects. K and TOF values of ADC-AuNPs, ADC-AgNPs and ADC-PdNPs are summarized and compared in Table 1. When 2.0 mol% catalysts were used, the 4-nitrophenol reduction was completed rapidly. At this point, ADC-AuNPs, ADC-AgNPs and ADC-PdNPs had K values of 2.16 × 10^−2^ s^−1^ (TOF: 900 h^−1^), 1.13 × 10^−2^ s^−1^ (TOF: 750 h^−1^), 2.60 × 10^−2^ s^−1^ (TOF: 1130 h^−1^), respectively. These results confirmed that ADC-PdNPs possessed the best catalytic activity. Furthermore, when the catalysts were reduced to 0.5 mol%, the same phenomenon was found. ADC-PdNPs (0.5 mol%) could accomplish the 4-nitrophenol reduction within 350 s with K and TOF values of 1.11 × 10^−2^ s^−1^ and 2060 h^−1^, ADC-AuNPs could complete the 4-nitrophenol reduction within 600 s, with K and TOF values of 6.70 × 10^−3^ s^−1^ and 1200 h^−1^, while ADC-AgNPs finished the 4-nitrophenol reduction in a longer time (1300 s), with relatively low K and TOF values of 4.50 × 10^−3^ s^−1^ and 550 h^−1^. As a consequence, ADC-PdNPs showed relatively higher catalytic activity in the 4-nitrophenol reduction than ADC-AuNPs and ADC-AgNPs. The good catalytic activity of ADC-PdNPs could be attributed to its small sizes, approximately monodisperse morphologies (Figure 4g–i), and the intrinsic stability of the Pd species [45]. The catalytic ability of ADC-PdNPs was also compared with the published literature in the 4-nitrophenol reduction. As shown in Table 2, the k_a_ and TOF values of relevant studies are summarized. The comparison clearly indicates that the current ADC-PdNPs had comparable catalytic activity, with a TOF value of up to 2060 h^−1^ in a relatively low dosage (0.5 mol%).

**Figure 6:**
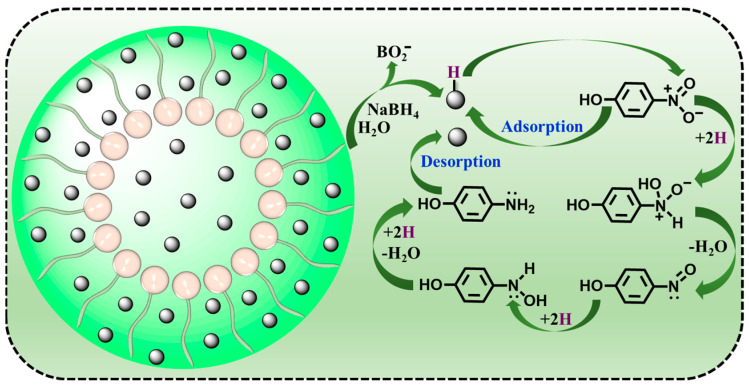
A possible reaction mechanism for the 4-nitrophenol reduction by ADC-encapsulated metal nanoparticles.

The possible reaction mechanism for the 4-nitrophenol reduction by ADC-encapsulated metal nanoparticles in the presence of NaBH_4_ was also proposed. Many previous studies have demonstrated that the 4-nitrophenol reduction mechanism using metal nanoparticle catalysts with the help of NaBH_4_ conforms to a Langmuir–Hinshelwood mechanism (Figure 6) [58,59,60,61,62]. The 4-nitrophenol reduction is completed on the metal nanoparticle surface via a hydrogen transfer process. Firstly, the hydrolysis of NaBH_4_ can generate BO^2−^ and active H_2_. Then, H_2_ is adsorbed into the metal nanoparticle surface, resulting in the formation of metal hydride (metal−H) intermediates. At the same time, 4-nitrophenol is deprotonated to produce a 4-nitrophenolate ion that is also adsorbed into the metal nanoparticle surface. Metal nanoparticles have the ability to accelerate the hydrolysis of NaBH_4_, thus promoting the metal−H generation. Subsequently, the metal−H intermediates are able to attack -NO_2_ groups, which results in the fast reduction of -NO_2_ to -NH_2_. In the 4-nitrophenol reduction, three consecutive hydro-deoxygenation procedures occur. The first process is that -NO_2_ groups are converted to -NO groups accompanied by dehydration, and the second process is that -NO groups are subjected to hydrogenation to produce -NHOH groups. The third process is that -NHOH groups suffer from further hydrogenation to generate -NH_2_ groups, which is proved to become the rate-determination step. Eventually, 4-aminophenol is desorbed from the metal nanoparticle surface, leading to a free surface. As a result, the metal nanoparticle catalyst can be regenerated and cycled.

Pd-based catalysts are considered to be the most common species for accelerating Suzuki−Miyaura reactions because of their high activity. In recent years, Pd nanoparticles have developed into efficient catalysts for Suzuki−Miyaura reactions [63,64,65]. Therefore, the catalytic activity of ADC-PdNPs is further investigated in Suzuki−Miyaura reactions. In order to optimize the reaction conditions, a model reaction between phenyl boronic acid and bromobenzene was carried out in the EtOH/H_2_O system. The parameters including the base and the catalyst loading were considered in the optimization process, as shown in Table 3. A diverse base (such as NaOH, K_3_PO_4_, KOH and K_2_CO_3_) was first used for the model reaction when 500 ppm ADC-PdNPs were employed as the catalyst (Table 3, entry 1–4). It was found that the isolated yields and TOF values were obtained at a relatively low level (yield: 48–69%; TOF: 95–138 h^−1^) using NaOH, K_3_PO_4_ and KOH as the base. Fortunately, the Suzuki−Miyaura reaction gave a high isolated yield (99%) and TOF (198 h^−1^) using K_2_CO_3_ as a base. As a consequence, K_2_CO_3_ was selected for further optimization reactions. The catalyst loading was gradually decreased from 500 ppm to 10 ppm. When 50–400 ppm catalysts were used for the Suzuki−Miyaura reaction, high isolated yields (>97%) could be achieved, and a high TOF value (1940 h^−1^) was also obtained (Table 3, entry 5–9). When the catalyst loading was reduced to 10 ppm, the Suzuki−Miyaura reaction might still have reached a 77% isolated yield with a high TOF value of 3850 h^−1^ but in a longer reaction time (20 h) (Table 3, entry 10). These results prove the high catalytic ability of ADC-PdNPs in the Suzuki−Miyaura reaction. Considering the isolated yields and reaction time, the optimum reaction conditions for the Suzuki−Miyaura reaction were labeled as a 50 pm catalyst and K_2_CO_3_ as the base.

The substrate scope of ADC-PdNPs was further evaluated in different substrates, as shown in Table 4. It was observed that all substrates containing electron-releasing or electron-withdrawing substituents could be translated into the desired biphenyl compounds in high isolated yields (91–99%) and TOF values (1820–1980 h^−1^). The chemical structures of these desired biphenyl compounds were measured by ^1^H NMR (Figures S1–S9). As a result, ADC-PdNPs were highly active for Suzuki–Miyaura reactions and had good applicability for different substrates.

The catalytic ability of ADC-PdNPs in the Suzuki–Miyaura reaction was also compared with published studies. As can be seen from Table 5, the catalyst loading, diameter, yield and TOF values of relevant studies were summarized based on different substrates. The comparison results distinctly demonstrated that ADC-PdNPs possessed superior qualities including small sizes (2.5 ± 1.0 nm), low catalyst loading (50 ppm), high yield (99%) and high TOF (1980 h^−1^).

**Figure 7:**
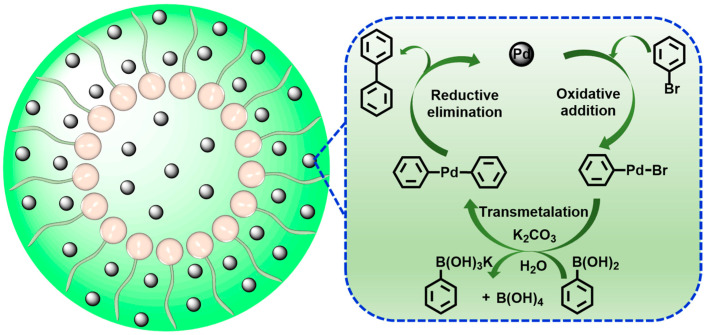
A possible reaction mechanism for Suzuki–Miyaura reduction.

The potential reaction mechanism for the Suzuki–Miyaura reaction via ADC-PdNPs is proposed. Many previous studies have discussed the mechanism of Pd-catalyzed Suzuki–Miyaura reactions [77,78,79,80]. As shown in Figure 7, the reaction process mainly involves three steps: oxidative addition, transmetalation and reductive elimination. In the oxidative addition step, aryl halide would oxidize Pd(0) to form σ-arylpalladium intermediate, which is considered as the rate-determining step. The σ-arylpalladium compound is able to react with aryl boronic acid to generate a biaryl palladium complex in the presence of K_2_CO_3_ through a transmetalation step. Finally, the biaryl palladium complex undergoes a reductive elimination to release the desired biaryl product. Meanwhile, the active Pd(0) catalyst was rebuilt for the next catalysis.

## 4. Conclusions

In summary, the ADC polymer containing hydrophilic branched triethylene glycol moieties and hydrophobic branched ferrocenyl moieties was successfully synthesized by ROMP and was applied as the supporting material to prepare metal nanoparticle catalysts including ADC-AuNPs, ADC-AgNPs and ADC-PdNPs. ADC-AuNPs, ADC-AgNPs and ADC-PdNPs had average sizes of 3.5 ± 3.0 nm, 7.2 ± 4.0 nm and 2.5 ± 1.0 nm, respectively, and showed quasi-spherical morphologies and a clear lattice fringe. The catalytic activities of ADC-AuNPs, ADC-AgNPs and ADC-PdNPs were investigated in the reduction of 4-nitrophenol. It was found that ADC-PdNPs exhibited relatively higher catalytic activity (K: 1.11 × 10^−2^ s^−1^; TOF: 2060 h^−1^) in the 4-nitrophenol reduction than ADC-AuNPs and ADC-AgNPs. In addition, ADC-PdNPs were also highly active for Suzuki–Miyaura reactions (low catalyst loading: 50 ppm; high yield: 99%; high TOF: 1980 h^−1^) and had good applicability for different substrates. Compared to existing catalysts, ADC-encapsulated metal NPs have the advantages of a high reaction rate constant, low catalyst dosage, high yields and high TOF values. The ADC polymer reported in this article gives a new inspiration for the design of the efficient supporting materials for metal nanoparticle catalysts.

## Figures and Tables

**Table 1:** 4-nitrophenol reduction with AuNPs, AgNPs or PdNPs.

Catalyst	Amount (mol%)	Diameter (nm)	K (s^−1^)	TOF (h^−1^)
AuNPs	2.0	3.5 ± 3.0	2.16 × 10^−2^	900
	0.5		6.70 × 10^−3^	1200
AgNPs	2.0	7.2 ± 4.0	1.13 × 10^−2^	750
	0.5		4.50 × 10^−3^	550
PdNPs	2.0	2.5 ± 1.0	2.60 × 10^−2^	1130
	0.5		1.11 × 10^−2^	2060

**Table 2:** Comparing the catalytic performance of ADC-PdNPs with published studies in the 4-nitrophenol reduction.

Catalyst	Amount	Diameter (nm)	K (s^−1^)	TOF (h^−1^)	Reference
Co_3_O_4_/HNTs	0.1 mg	9.4	4.42 × 10^−3^	262	[46]
AgPd-120	-	27.6 ± 3.1	4.07 × 10^−3^	765	[47]
Ag/ZnO	1 mg	-	4.92 × 10^−3^	-	[48]
Pd/Mo_2_N-TiO_2_	0.8 wt%	2.4 ± 0.33	1.96 × 10^−2^	698	[49]
Pd/3D-AC	0.1 g	2.97	1.50 × 10^−2^	101	[50]
AuAgPd PNFs	40 μg	-	1.04 × 10^−3^	-	[51]
Pd@CCTP	2.0 mg	20–25	2.08 × 10^−2^	883	[52]
Ag_0.5_/C15h	0.51 wt%	3.5 ± 1.1	1.22 × 10^−3^	-	[53]
Pd/TiO_2_	0.11 wt%	2.0–5.0	1.22 × 10^−2^	161	[54]
Pd/GO-P	0.00065 mg	3.9 ± 0.4	2.32 × 10^−2^	-	[55]
Cu/MXene/PAM	1.0 mg	-	1.26 × 10^−2^	-	[56]
UiO-66-NH_2_/Pd@HMSN	0.05 mg	5.07	9.24 × 10^−3^	214	[57]
ADC-PdNPs	2.0 mol%	2.5 ± 1.0	2.60 × 10^−2^	1130	This work
ADC-PdNPs	0.5 mol%	2.5 ± 1.0	1.11 × 10^−2^	2060	This work

**Table 3:** Optimizing Suzuki–Miyaura reaction using ADC-PdNPs as the catalyst.

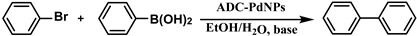					
Entry	Catalyst (ppm)	Base	Time (h)	Isolated Yield (%)	TOF (h^−1^)
1	500	NaOH	10	66	132
2	500	K_3_PO_4_	10	48	96
3	500	KOH	10	69	138
4	500	K_2_CO_3_	10	99	325 ^a^
5	400	K_2_CO_3_	10	99	394 ^a^
6	300	K_2_CO_3_	10	99	483 ^a^
7	200	K_2_CO_3_	10	99	700 ^a^
8	100	K_2_CO_3_	10	99	1257 ^a^
9	50	K_2_CO_3_	10	97	2250 ^a^
10	10	K_2_CO_3_	20	77	3850

Reaction conditions: bromobenzene (1.0 mmol), phenylboronic acid (1.2 mmol), ADC-PdNPs, base (2.0 mmol), EtOH (2.5 mL)/H_2_O (2.5 mL), 85 °C. ^a^ TOF was calculated after 4 h.

**Table 4:** Catalytic activities of ADC-PdNPs in Suzuki–Miyaura reactions based on all kinds of substrates.

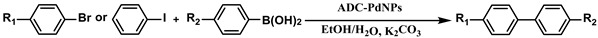				
Entry	Aryl Halide	Aryl Boronic Acid	Isolated Yield (%)	TOF (h^−1^)
1	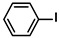	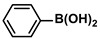	99	1980
2	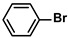	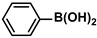	97	1940
3	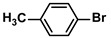	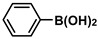	95	1900
4	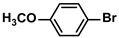	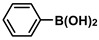	96	1920
5	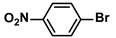	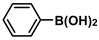	93	1860
6	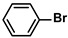	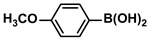	96	1920
7	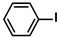	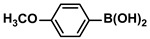	98	1960
8	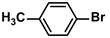	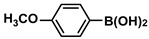	94	1880
9	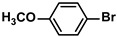	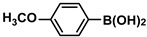	95	1900
10	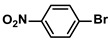	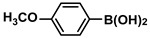	92	1840
11	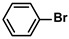	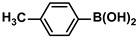	91	1820
12	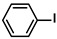	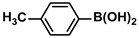	96	1920
13	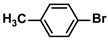	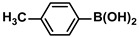	92	1840
14	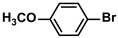	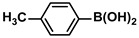	95	1900
15	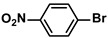	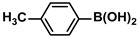	93	1860

Reaction conditions: aryl halide (1.0 mmol), aryl boronic acid (1.2 mmol), ADC-PdNPs (50 ppm), K_2_CO_3_ (2.0 mmol), EtOH (2.0 mL)/H_2_O (2.0 mL), 10 h, 85 °C.

**Table 5:** Comparing the catalytic performance of ADC-PdNPs in Suzuki–Miyaura reactions with published studies.

Catalyst	Amount	Aryl Halide	Aryl Boronic Acid	Diameter (nm)	Yield (%)	TOF (h^−1^)	Reference
Fe_3_O_4_/SiO_2_-NH_2_@CS/Pd	0.1 mol%	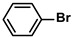	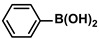	5.0	98	980	[66]
Pd–Ag@PMFC	0.008 g	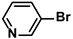	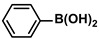	52.0	93	-	[67]
Fe_3_O_4_@H_2_L-Pd(0)	0.8 mol%	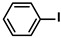	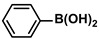	12.0–19.0	99	-	[68]
DMSTNs-SH-Pd	0.2 mol%	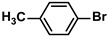	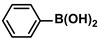	3.1	78	780	[69]
Pd@InOF-1	10.0 mg	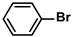	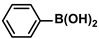	3.14	94	-	[70]
Fe_3_O_4_@SiO_2_-DTPA-Pd	1.7 µmol	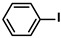	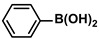	-	95	186	[71]
g-CN(G)-AgPd	5.0 mg	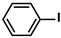	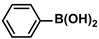	2.54 ± 0.76	94	-	[72]
PAF-SP@Pd	20 mg	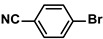	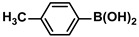	1–10	98	12	[73]
Pd/COF-SMC_2_	0.5 mol %	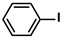	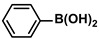	5	96	192	[74]
Cu/Pd@Mod-PANI-3OH	0.094 mol%	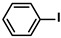	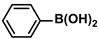	<1	93	659	[75]
SBA-15/Et-CN/Pd	0.1 mol%	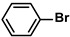	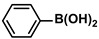	-	95	950	[76]
PdNPs	50 ppm	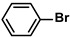	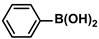	2.0 ± 1.0	98	2450	This work

## Data Availability

Data are contained within the article and Supplementary Materials.

## Supplementary Materials

The following supporting information can be downloaded at: https://www.mdpi.com/article/10.3390/polym16081080/s1, Figure S1: 1H NMR spectra of 1,1′-biphenyl; Figure S2: 1H NMR spectra of 4-methyl-1,1′-biphenyl; Figure S3: 1H NMR spectra of 4-methoxy-1,1′-biphenyl; Figure S4: 1H NMR spectra of 4-nitro-1,1′-biphenyl; Figure S5: 1H NMR spectra of 4-methoxy-4′-methyl-1,1′-biphenyl; Figure S6: 1H NMR spectra of 4,4′-dimethoxy-1,1′-biphenyl; Figure S7: 1H NMR spectra of 4-methoxy-4′-nitro-1,1′-biphenyl; Figure S8: 1H NMR spectra of 4,4′-dimethyl-1,1′-biphenyl; Figure S9: 1H NMR spectra of 4-methyl-4′-nitro-1,1′-biphenyl.

## References

[1] Gao C.B., Lyu F.L., Yin Y.D. (2021). Encapsulated Metal Nanoparticles for Catalysis. Chem. Rev..

[2] Wang Q., Astruc D. (2020). State of the Art and Prospects in Metal–Organic Framework (MOF)-Based and MOF-Derived Nanocatalysis. Chem. Rev..

[3] Axet M.R., Philippot K. (2020). Catalysis with Colloidal Ruthenium Nanoparticles. Chem. Rev..

[4] Trzeciak A.M., Augustyniak A.W. (2019). The role of palladium nanoparticles in catalytic C–C cross-coupling reactions. Coord. Chem. Rev..

[5] Shifrina Z.B., Matveeva V.G., Bronstein L.M. (2020). Role of Polymer Structures in Catalysis by Transition Metal and Metal Oxide Nanoparticle Composites. Chem. Rev..

[6] Sun X.Y., Li S.F., Cao J.Z., Wang Y.C., Yang W.B., Zhang L.J., Liu Y.J., Qiu J.S., Tao S.Y. (2021). A Hierarchical-Structured Impeller with Engineered Pd Nanoparticles Catalyzing Suzuki Coupling Reactions for High-Purity Biphenyl. ACS Appl. Mater. Interfaces.

[7] Xu Y.L., Shi X.F., Hua R., Zhang R., Yao Y.J., Zhao B., Liu T., Zheng J.Z., Lu G. (2020). Remarkably catalytic activity in reduction of 4-nitrophenol and methylene blue by Fe_3_O_4_@COF supported noble metal nanoparticles. Appl. Catal. B Environ..

[8] Wang J.C., Liu C.X., Kan X., Wu X.W., Kan J.L., Dong Y.B. (2020). Pd@COF-QA: A phase transfer composite catalyst for aqueous Suzuki–Miyaura coupling reaction. Green Chem..

[9] Favier I., Pla D., Gomez M. (2020). Palladium Nanoparticles in Polyols: Synthesis, Catalytic Couplings, and Hydrogenations. Chem. Rev..

[10] Shi Y.F., Lyu Z.H., Zhao M., Chen R.H., Nguyen Q.N., Xia Y.N. (2021). Noble-Metal Nanocrystals with Controlled Shapes for Catalytic and Electrocatalytic Applications. Chem. Rev..

[11] Gan W.P., Xu H., Jin X.Y., Cao X.S., Gao H.F. (2020). Recyclable Palladium-Loaded Hyperbranched Polytriazoles as Efficient Polymer Catalysts for Heck Reaction. ACS Appl. Energy Mater..

[12] Zhang W.J., Kong H.M., Wu Z.N., Yao A.F., Wang L.A., Quo L., He Y.J., Qiao X.G., Pang X.C., Xie J.P. (2021). Confined Unimolecular Micelles for Precisely Controlled In Situ Synthesis of Stable Ultrasmall Metal Nanocluster Assemblies. Chem. Mater..

[13] Huang T.F., Sheng G., Manchanda P., Emwas A.H., Lai Z.P., Nunes S.P., Peinemann K.V. (2019). Cyclodextrin polymer networks decorated with subnanometer metal nanoparticles for high-performance low-temperature catalysis. Sci. Adv..

[14] Frauenrath H. (2005). Dendronized polymers—Building a new bridge from molecules to nanoscopic objects. Prog. Polym. Sci..

[15] Liu X., Liu F.F., Liu W.T., Gu H.B. (2020). ROMP and MCP as Versatile and Forceful Tools to Fabricate Dendronized Polymers for Functional Applications. Polym. Rev..

[16] Chen Y.M., Xiong X.Q. (2010). Tailoring dendronized polymers. Chem. Commun..

[17] Liang C.O., Helms B., Hawker C.J., Fréchet J.M.J. (2003). Dendronized cyclocopolymers with a radial gradient of polarity and their use to catalyze a difficult esterification. Chem. Commun..

[18] Liu X., Lin W., Astruc D., Gu H.B. (2019). Syntheses and applications of dendronized polymers. Prog. Polym. Sci..

[19] Liu F.F., Liu X., Astruc D., Gu H.B. (2019). Dendronized triazolyl-containing ferrocenyl polymers as stabilizers of gold nanoparticles for recyclable two-phase reduction of 4-nitrophenol. J. Colloid Interface Sci..

[20] Lu J.H., Yang Y., Gao J.F., Duan H.C., Lu C.L. (2018). Thermoresponsive Amphiphilic Block Copolymer-Stablilized Gold Nanoparticles: Synthesis and High Catalytic Properties. Langmuir.

[21] Liu X., Liu F.F., Astruc D., Lin W., Gu H.B. (2019). Highly-branched amphiphilic organometallic dendronized diblock copolymer: ROMP synthesis, self-assembly and long-term Au and Ag nanoparticle stabilizer for high-efficiency catalysis. Polymer.

[22] Liu X., Ling Q.J., Zhao L., Qiu G.R., Wang Y.H., Song L.X., Zhang Y., Ruiz J., Astruc D., Gu H.B. (2017). New ROMP synthesis of ferrocenyl dendronized polymers. Macromol. Rapid Commun..

[23] Liu X., Qiu G.R., Zhang L., Liu F.F., Mu S.D., Long Y.R., Zhao Q.X., Liu Y., Gu H.B. (2018). Controlled ROMP Synthesis of Ferrocene-Containing Amphiphilic Dendronized Diblock Copolymers as Redox-Controlled Polymer Carriers. Macromol. Chem. Phys..

[24] Ogba O.M., Warner N.C., O’Leary D.J., Grubbs R.H. (2018). Recent Advances in Ruthenium-Based Olefin Metathesis. Chem. Soc. Rev..

[25] Liu X., Rapakousiou A., Deraedt C., Ciganda R., Wang Y.L., Ruiz J., Gu H.B., Astruc D. (2020). Multiple Applications of Polymers Containing Electron-reservoir Metal-sandwich Complexes. Chem. Commun..

[26] Liu X., Ren Z.J., Liu F.F., Zhao L., Ling Q.J., Gu H.B. (2021). Multifunctional Self-Healing Dual Network Hydrogels Constructed via Host–Guest Interaction and Dynamic Covalent Bond as Wearable Strain Sensors for Monitoring Human and Organ Motions. ACS Appl. Mater. Interfaces.

[27] Liu F.F., Liu X., Gu H.B. (2022). Multi-Network Poly(β-cyclodextrin)/PVA/Gelatin/Carbon Nanotubes Composite Hydrogels Constructed by Multiple Dynamic Crosslinking as Flexible Electronic Devices. Macromol. Mater. Eng..

[28] Deraedt C., Salmon L., Etienne L., Ruiz J., Astruc D. (2013). “Click” dendrimers as efficient nanoreactors in aqueous solvent: Pd nanoparticle stabilization for sub-ppm Pd catalysis of Suzuki-Miyaura reactions of aryl bromides. Chem. Commun..

[29] Wang C.L., Ciganda R., Salmon L., Gregurec D., Irigoyen J., Moya S., Ruiz J., Astruc D. (2016). Highly Efficient Transition Metal Nanoparticle Catalysts in Aqueous Solutions. Angew. Chem. Int. Ed..

[30] Huang T., Meng F., Qi L.M. (2009). Facile Synthesis and One-Dimensional Assembly of Cyclodextrin-Capped Gold Nanoparticles and Their Applications in Catalysis and Surface-Enhanced Raman Scattering. J. Phys. Chem. C.

[31] Yoshida H., Kuwauchi Y., Jinschek J.R., Sun K.J., Tanaka S., Kohyama M., Shimada S., Haruta M., Takeda S. (2012). Visualizing Gas Molecules Interacting with Supported Nanoparticulate Catalysts at Reaction Conditions. Science.

[32] Li J., Liu C.Y., Liu Y. (2012). Au/graphene hydrogel: Synthesis, characterization and its use for catalytic reduction of 4-nitrophenol. J. Mater. Chem..

[33] Yuwen L.H., Xu F., Xue B., Luo Z.M., Zhang Q., Bao B.Q., Su S., Weng L.X., Huang W., Wang L.H. (2014). General synthesis of noble metal (Au, Ag, Pd, Pt) nanocrystal modified MoS2 nanosheets and the enhanced catalytic activity of Pd–MoS2 for methanol oxidation. Nanoscale.

[34] Ogasawara S., Kato S. (2010). Palladium Nanoparticles Captured in Microporous Polymers: A Tailor-Made Catalyst for Heterogeneous Carbon Cross-Coupling Reactions. J. Am. Chem. Soc..

[35] Hong J.W., Lee Y.W., Kim M., Kang S.W., Han S.W. (2011). One-pot synthesis and electrocatalytic activity of octapodal Au-Pd nanoparticles. Chem. Commun..

[36] Hervés P., Pérez-Lorenzo M., Liz-Marzán L.M., Dzubiella J., Lubc Y., Ballauff M. (2012). Catalysis by metallic nanoparticles in aqueous solution: Model reactions. Chem. Soc. Rev..

[37] Jiang H.L., Akita T., Ishida T., Haruta M., Xu Q. (2011). Synergistic catalysis of Au@Ag core-shell nanoparticles stabilized on metal-organic framework. J. Am. Chem. Soc..

[38] Zeng J., Zhang Q., Chen J.Y., Xia Y.N. (2010). A comparison study of the catalytic properties of Au-based nanocages, nanoboxes, and nanoparticles. Nano Lett..

[39] Gangula A., Podila R., Karanam L., Janardhana C., Rao A.M. (2011). Catalytic reduction of 4-nitrophenol using biogenic gold and silver nanoparticles derived from Breynia rhamnoides. Langmuir.

[40] Li H., Han L., Cooperwhite J., Kim I. (2012). Palladium nanoparticles decorated carbon nanotubes: Facile synthesis and their applications as highly efficient catalysts for the reduction of 4-nitrophenol. Green Chem..

[41] Emmanuel R., Karuppiah C., Chen S.M., Palanisamy S., Padmavathy S., Prakash P. (2014). Green synthesis of gold nanoparticles for trace level detection of a hazardous pollutant (nitrobenzene) causing Methemoglobinaemia. J. Hazard. Mater..

[42] Nemanashi M., Meijboom R. (2013). Synthesis and characterization of Cu, Ag and Au dendrimer-encapsulated nanoparticles and their application in the reduction of 4-nitrophenol to 4-aminophenol. J. Colloid Interface Sci..

[43] Liu F.F., Liu X., Chen F., Fu Q. (2021). Tannic Acid: A green and efficient stabilizer of Au, Ag, Cu and Pd nanoparticles for the 4-Nitrophenol Reduction, Suzuki-Miyaura coupling reactions and click reactions in aqueous solution. J. Colloid Interface Sci..

[44] Lan T.X., An R., Liu Z., Li K.J., Xiang J., Liu G.Y. (2018). Facile fabrication of a biomass-based film with interwoven fibrous network structure as heterogeneous catalysis platform. J. Colloid Interface Sci..

[45] Jin Z., Xiao M., Bao Z., Wang P., Wang J. (2012). A General Approach to Mesoporous Metal Oxide Microspheres Loaded with Noble Metal Nanoparticles. Angew. Chem. Int. Edit..

[46] Zhang M., Su X.T., Ma L.D., Khan A., Wang L., Wang J.D., Maloletnev A.S., Yang C. (2021). Promotion effects of halloysite nanotubes on catalytic activity of Co3O4 nanoparticles toward reduction of 4-nitrophenol and organic dyes. J. Hazard. Mater..

[47] Ma W.J., Zhang G.M., Zhang P., Fu Z.Y. (2022). Ag-Pd bimetallic hollow nanostructures with tunable compositions and structures for the reduction of 4-nitrophenol. J. Alloys Compd..

[48] Hunge Y.M., Yadav A.A., Kang S.W., Kim H. (2022). Facile synthesis of multitasking composite of Silver nanoparticle with Zinc oxide for 4-nitrophenol reduction, photocatalytic hydrogen production, and 4-chlorophenol degradation. J. Alloys Compd..

[49] Tian X.Q., Muhammad Z., Li J., Sun W., Niu X.Y., Zhu Y.J. (2020). Pd/Mo_2_N-TiO_2_ as efficient catalysts for promoted selective hydrogenation of 4-nitrophenol: A green bio-reducing preparation method. J. Catal..

[50] Shu F., Wu J., Jiang G.P., Qiao Y.Z., Wang Y.L., Wu D.D., Zhong Y.J., Zhang T.W., Song J.L., Jin Y.C. (2022). A hierarchically porous and hygroscopic carbon-based catalyst from natural wood for efficient catalytic reduction of industrial high-concentration 4-nitrophenol. Sep. Purif. Technol..

[51] Kong Y.H., Sun Q.X., Zhang G.G., Liu F., Liu M.C., Zheng Y.Q. (2022). Stepwise synthesis of polyhedral AuAgPd nanoframes for plasmon-enhanced catalytic reduction of 4-nitrophenol. Mater. Lett..

[52] Yadav D., Awasthi S.K. (2020). A Pd confined hierarchically conjugated covalent organic polymer for hydrogenation of nitroaromatics: Catalysis, kinetics, thermodynamics and mechanism. Green Chem..

[53] Chen C.S., Chen T.C., Chiu K.L., Wu H.C., Pao C.W., Chen C.L., Hsu H.C., Kao H.M. (2022). Silver particles deposited onto magnetic carbon nanofibers as highly active catalysts for 4-nitrophenol reduction. Appl. Catal. B Environ..

[54] Liu T., Sun Y.H., Jiang B., Guo W., Qin W., Xie Y.M., Zhao B., Zhao L., Liang Z.Q., Jiang L. (2020). Pd Nanoparticle-Decorated 3D-Printed Hierarchically Porous TiO2 Scaffolds for the Efficient Reduction of a Highly Concentrated 4-Nitrophenol Solution. ACS Appl. Mater. Interfaces.

[55] Zhang T.D., Ouyang B., Zhang X.L., Xia G.Q., Wang N.T., Ou H.Y., Ma L., Mao P.X., Ostrikov K.K., Di L.B. (2022). Plasma-enabled synthesis of Pd/GO rich in oxygen-containing groups and defects for highly efficient 4-nitrophenol reduction. Appl. Surf. Sci..

[56] Li X.Z., Peng W., Li L., Chen S., Ye L., Peng C. (2022). Simple synthesis of copper/MXene/polyacrylamide hydrogel catalyst for 4-nitrophenol reduction. Mater. Lett..

[57] Huang X.J., Lin D.Y., Duan P., Chen H.P., Zhao Y.J., Yang W.T., Pan Q.H., Tian X.L. (2023). Space-confined growth of nanoscale metal-organic frameworks/Pd in hollow mesoporous silica for highly efficient catalytic reduction of 4-nitrophenol. J. Colloid Interface Sci..

[58] Wang Q.L., Wei Z.J., Li J., Feng D.Y., Feng A., Zhang H. (2022). Hierarchical-Structured Pd Nanoclusters Catalysts x-PdNCs/CoAl(O)/rGO-T by the Captopril-Capped Pd Cluster Precursor Method for the Highly Efficient 4-Nitrophenol Reduction. ACS Appl. Mater. Interfaces.

[59] Feng D.Y., Wei Z.J., Wang Q.L., Feng A., Zhang H. (2022). Controllable Synthesis of Cobalt-Containing Nanosheet Array-Like Ternary CuCoAl-LDH/rGO Hybrids to Boost the Catalytic Efficiency for 4-Nitrophenol Reduction. ACS Appl. Mater. Interfaces.

[60] Wunder S., Lu Y., Albrecht M., Ballauff M. (2011). Catalytic Activity of Faceted Gold Nanoparticles Studied by a Model Reaction: Evidence for Substrate-Induced Surface Restructuring. ACS Catal..

[61] Ansar S.M., Kitchens C.L. (2016). Impact of Gold Nanoparticle Stabilizing Ligands on the Colloidal Catalytic Reduction of 4-Nitrophenol. ACS Catal..

[62] Wunder S., Polzer F., Lu Y., Mei Y., Ballauff M. (2010). Kinetic analysis of catalytic reduction of 4-nitrophenol by metallic nanoparticles immobilized in spherical polyelectrolyte brushes. J. Phys. Chem. C.

[63] Beletskaya I.P., Alonso F., Tyurin V. (2019). The Suzuki-Miyaura reaction after the Nobel prize. Coord. Chem. Rev..

[64] Biffis A., Centomo P., Del Zotto A., Zecca M. (2018). Pd Metal catalysts for cross-couplings and related reactions in the 21st century: A critical review. Chem. Rev..

[65] Fihri A., Bouhrara M., Nekoueishahraki B., Basset J.M., Polshettiwar V. (2011). Nanocatalysts for Suzuki cross-coupling reactions. Chem. Soc. Rev..

[66] Veisi H., Ozturk T., Karmakar B., Tamoradi T., Hemmati S. (2020). In situ decorated Pd NPs on chitosan-encapsulated Fe_3_O_4_/SiO_2_-NH_2_ as magnetic catalyst in Suzuki-Miyaura coupling and 4-nitrophenol reduction. Carbohydr. Polym..

[67] Bhattacharjee P., Dewan A., Boruah P.K., Das M.R., Mahanta S.P., Thakur A.J., Bora U. (2022). Bimetallic Pd–Ag nanoclusters decorated micro-cellulose bio-template towards efficient catalytic Suzuki–Miyaura coupling reaction of nitrogen-rich heterocycles. Green Chem..

[68] Jasim S.A., Ansari M.J., Majdi H.S., Opulencia M.J.C., Uktamov K.F. (2022). Nanomagnetic Salamo-based-Pd(0) Complex: An efficient heterogeneous catalyst for Suzuki–Miyaura and Heck cross-coupling reactions in aqueous medium. J. Mol. Struct..

[69] Wang Y.B., Tao J.H., Wang Y.N., Huang L.Z., Ding X.P. (2022). Remarkable reduction ability towards p-nitrophenol by a synergistic effect against the aggregation and leaching of palladium nanoparticles in dendritic supported catalysts. Appl. Surf. Sci..

[70] Chen D.D., Wei L.S., Yu Y.H., Zha L., Sun Q.H., Han C., Lu J.M., Nie H.G., Shao L.X., Qian J.J. (2022). Size-Selective Suzuki–Miyaura Coupling Reaction over Ultrafine Pd Nanocatalysts in a Water-Stable Indium–Organic Framework. Inorg. Chem..

[71] Ren F.F., Li S.M., Zheng W.Q., Song Q.Y., Jia W.H., Nan Y.Q., Jia H.J., Liu J., Li Y.X. (2022). Preparation of a novel heterogeneous palladium nanocatalyst based on carboxyl modified magnetic nanoparticles and its applications in Suzuki-Miyaura coupling reactions. Colloids Surf. A Physicochem. Eng. Asp..

[72] Altan O., Kalay E. (2022). The influence of band bending phenomenon on photocatalytic Suzuki-Miyaura coupling reaction: The case of AgPd alloy nanoparticles supported on graphitic carbon nitride. Appl. Surf. Sci..

[73] Căta L., Terenti N., Cociug C., Hădade N.D., Grosu I., Bucur C., Cojocaru B., Parvulescu V.I., Mazur M., Čejka J. (2022). Sonogashira Synthesis of New Porous Aromatic Framework-Entrapped Palladium Nanoparticles as Heterogeneous Catalysts for Suzuki–Miyaura Cross-Coupling. ACS Appl. Mater. Interfaces.

[74] Liu J.G., Zhan H., Wang N., Song Y.P., Wang C.G., Wang X.M., Ma L.L., Chen L.G. (2021). Palladium Nanoparticles on Covalent Organic Framework Supports as Catalysts for Suzuki–Miyaura Cross-Coupling Reactions. ACS Appl. Nano Mater..

[75] Wang G., Hao P.C., Chang Y.J., Zhang Q.P., Liu W.Y., Duan B., Zhan H.J., Bi S.X. (2022). Copper and palladium bimetallic sub-nanoparticles were stabilized on modified polyaniline materials as an efficient catalyst to promote C–C coupling reactions in aqueous solution. Nanoscale.

[76] Wang J.L., Wang L.L., Cai X.P., Karmakar B., Zangeneh M.M., Liu H.R. (2021). Pd nanoparticles fabricated cyano-functionalized mesoporous SBA-15: A novel heterogeneous catalyst for Suzuki-Miyaura coupling reactions and anti-human lung cancer effects. Mater. Chem. Phys..

[77] Veisi H., Joshani Z., Karmakar B., Tamoradi T., Heravi M.M., Gholami J. (2021). Ultrasound assisted synthesis of Pd NPs decorated chitosan-starch functionalized Fe3O4 nanocomposite catalyst towards Suzuki-Miyaura coupling and reduction of 4-nitrophenol. Int. J. Biol. Macromol..

[78] Palem R.R., Shimoga G., Kim S.Y., Bathula C., Ghodake G.S., Lee S.H. (2022). Biogenic palladium nanoparticles: An effectual environmental benign catalyst for organic coupling reactions. J. Ind. Eng. Chem..

[79] Fan M.Y., Wang W.D., Wang X.Y., Zhu Y.Y., Dong Z.P. (2022). Ultrafine Pd Nanoparticles Modified on Azine-Linked Covalent Organic Polymers for Efficient Catalytic Suzuki–Miyaura Coupling Reaction. Ind. Eng. Chem. Res..

[80] Heidari F., Hekmati M., Veisi H. (2017). Magnetically separable and recyclable Fe_3_O_4_@SiO_2_/isoniazide/Pd nanocatalyst for highly efficient synthesis of biaryls by Suzuki coupling reactions. J. Colloid Interface Sci..

