# Supplementary material for: Amphiphilic Dendronized Copolymer-Encapsulated Au, Ag and Pd Nanoparticles for Catalysis in the 4-Nitrophenol Reduction and Suzuki–Miyaura Reactions
Source: Polymers (Basel). 2024 Apr 12;16(8):1080. doi: 10.3390/polym16081080 (PMC11054709; doi:10.3390/polym16081080)
Supplement: Supplementary file 1 [file polymers-16-01080-s001.zip › polymers-2942152-supplementary.pdf]

polymers

Supporting Information

Amphiphilic Dendronized Copolymer-Encapsulated Au, Ag and Pd Nanoparticles for Catalysis in the 4-Nitrophenol Reduction and Suzuki–Miyaura Reactions

Fangfei Liu and Xiong Liu *

State Key Laboratory of Chemistry and Utilization of Carbon Based Energy Resources; College of Chemistry, Xinjiang University, Urumqi, 830017, Xinjiang, PR China.

* Correspondence: liuxiong@xju.edu.cn

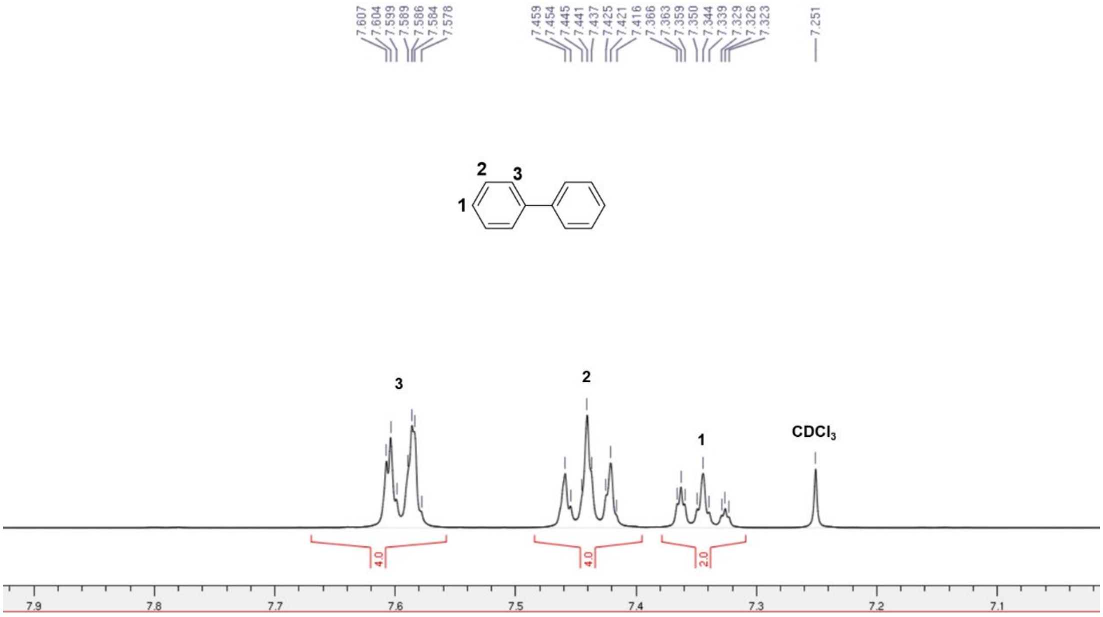

Figure S1. ^{1}H NMR spectra of 1,1'-biphenyl.

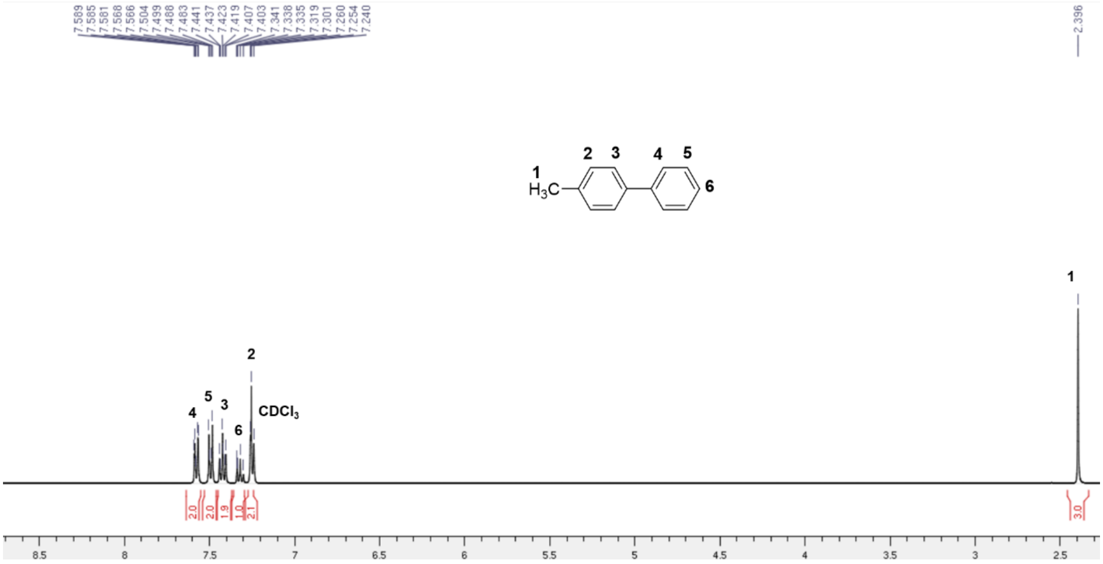

Figure S2. ^{1}H NMR spectra of 4-methyl-1,1'-biphenyl.

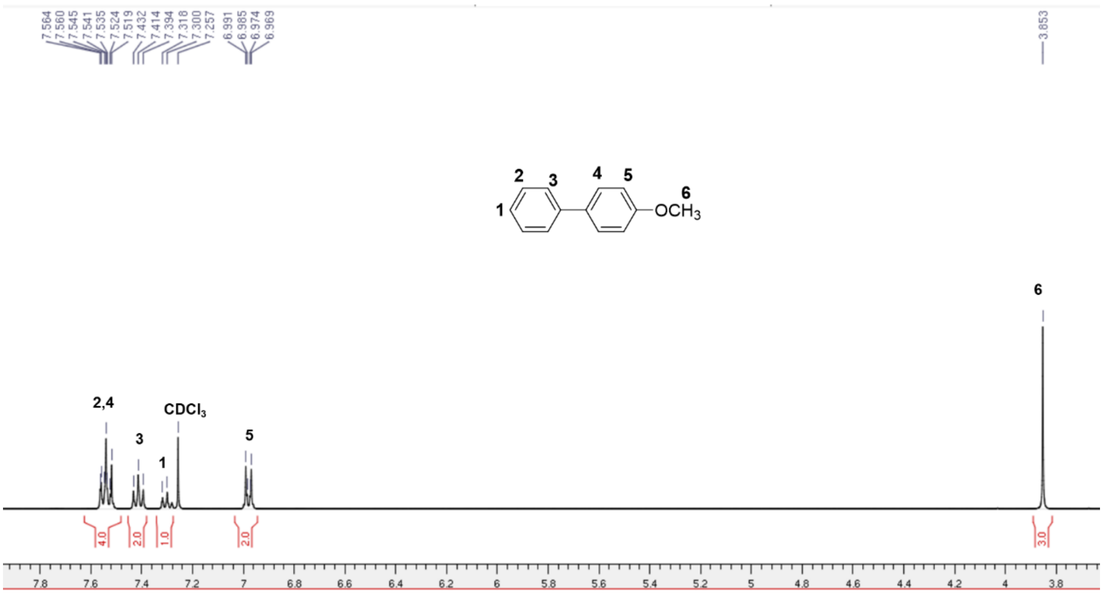

Figure S3. ^{1}H NMR spectra of 4-methoxy-1,1'-biphenyl.

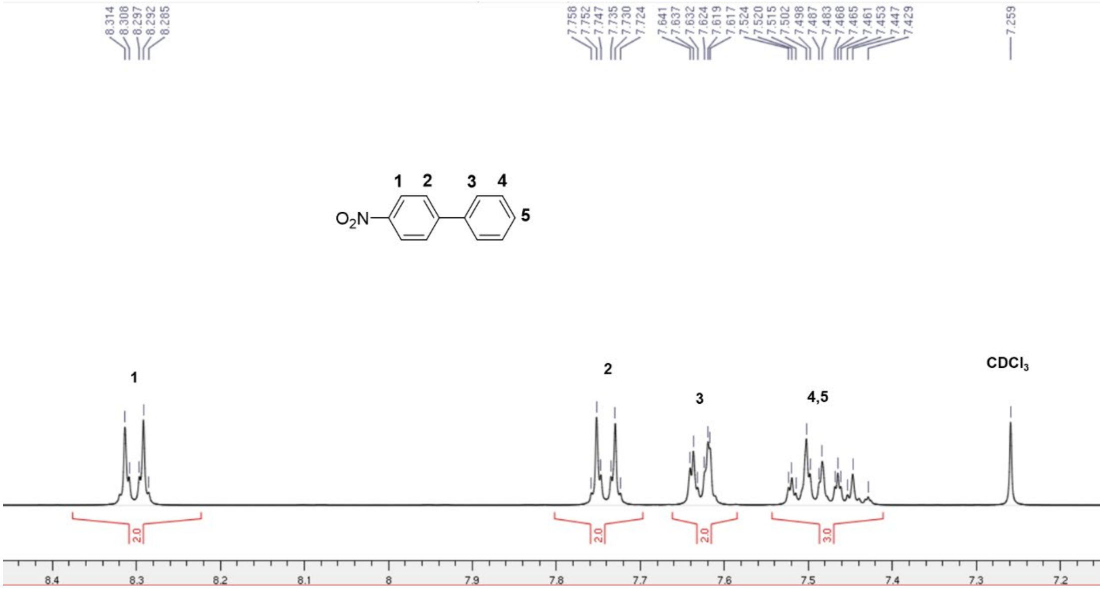

Figure S4. ^{1}H NMR spectra of 4-nitro-1,1'-biphenyl.

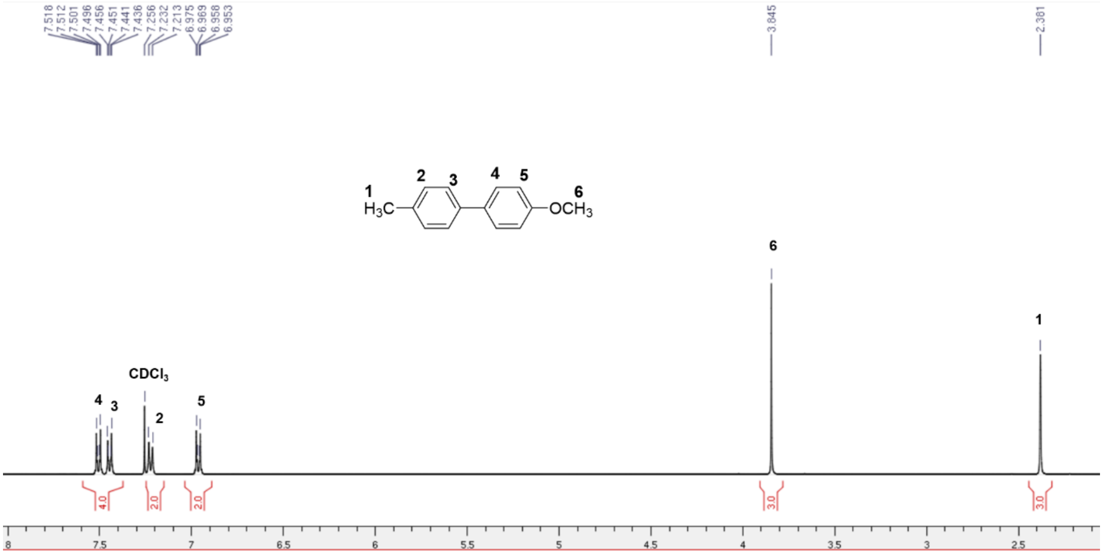

Figure S5. ^{1}H NMR spectra of 4-methoxy-4'-methyl-1,1'-biphenyl.

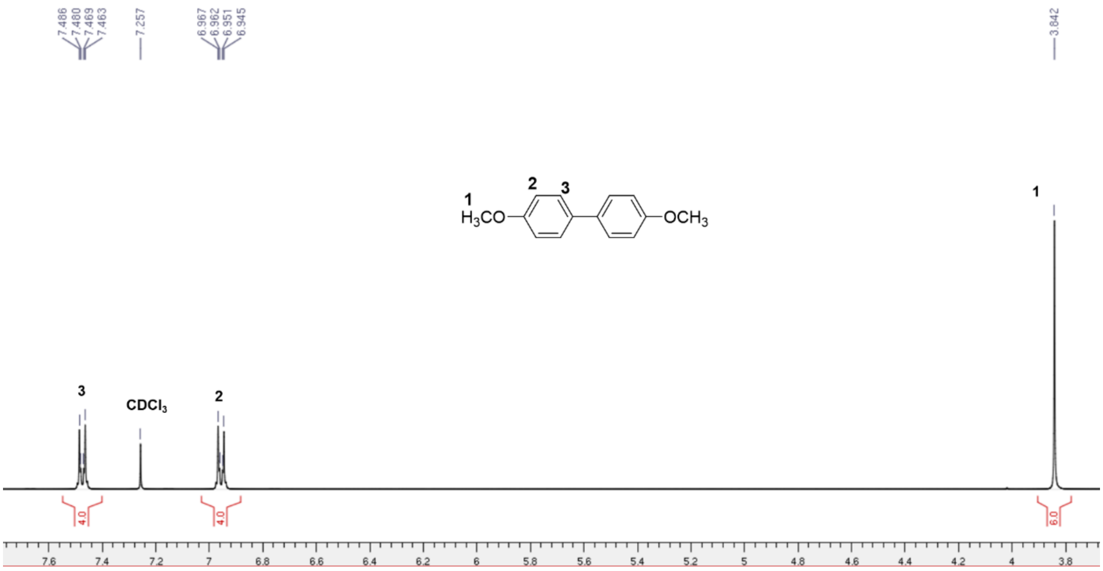

Figure S6. ^{1}H NMR spectra of 4,4'-dimethoxy-1,1'-biphenyl.

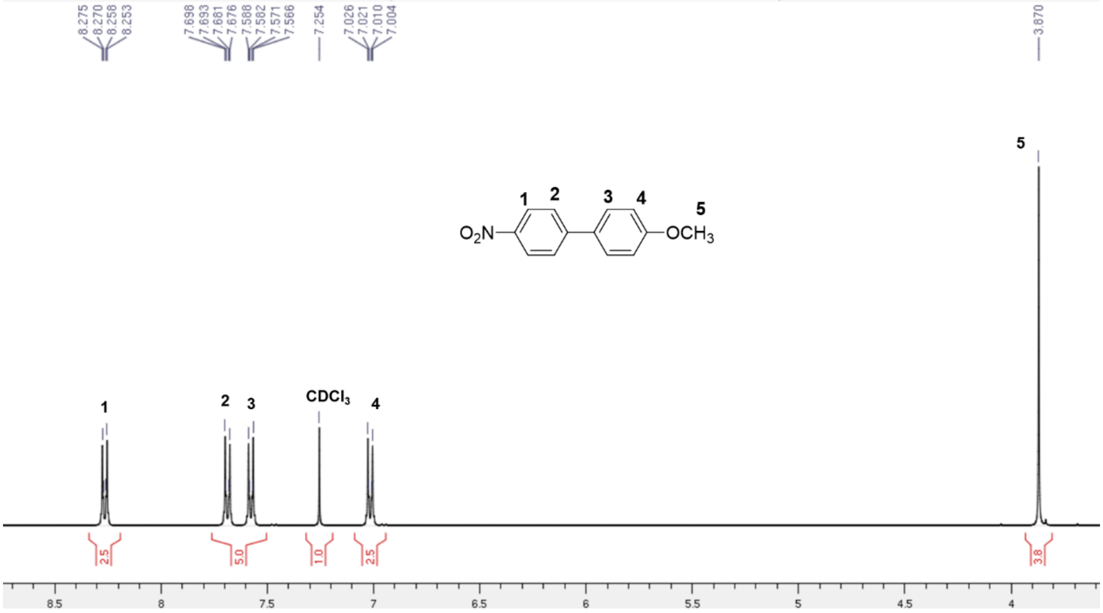

Figure S7. ^{1}H NMR spectra of 4-methoxy-4'-nitro-1,1'-biphenyl.

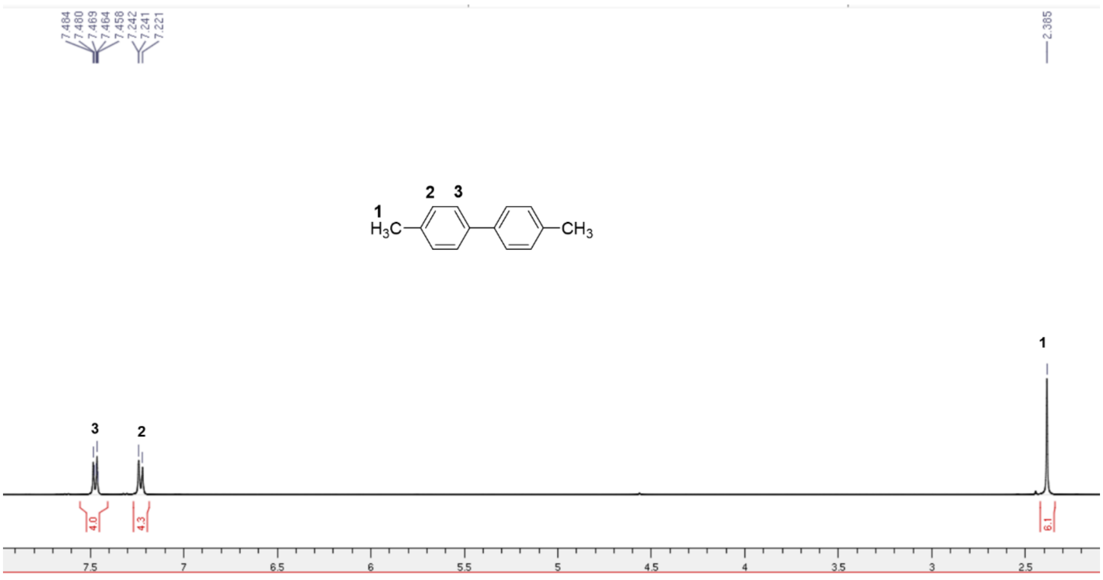

Figure S8. ^{1}H NMR spectra of 4,4'-dimethyl-1,1'-biphenyl.

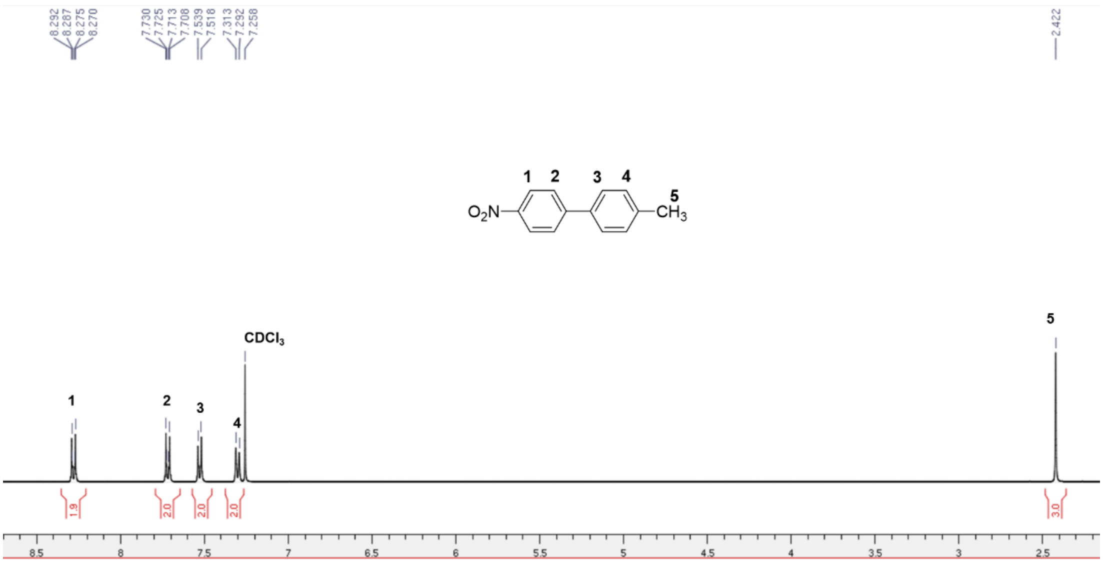

Figure S9. ^{1}H NMR spectra of 4-methyl-4'-nitro-1,1'-biphenyl.
